# Movement patterns of Tenebrio beetles demonstrate empirically that correlated-random-walks have similitude with a Lévy walk

**DOI:** 10.1038/srep03158

**Published:** 2013-11-07

**Authors:** Andy M. Reynolds, Lisa Leprêtre, David A. Bohan

**Affiliations:** 1 Rothamsted Research, Harpenden, Hertfordshire, AL5 2JQ, UK; 2 Université de Bourgogne, UMR CNRS 6282 Biogéosciences, F-21000 Dijon, France; 3 INRA, UMR1347 Agroécologie, BP 86510, F-21000 Dijon, France

## Abstract

Correlated random walks are the dominant conceptual framework for modelling and interpreting organism movement patterns. Recent years have witnessed a stream of high profile publications reporting that many organisms perform Lévy walks; movement patterns that seemingly stand apart from the correlated random walk paradigm because they are discrete and scale-free rather than continuous and scale-finite. Our new study of the movement patterns of *Tenebrio* *molitor* beetles in unchanging, featureless arenas provides the first empirical support for a remarkable and deep theoretical synthesis that unites correlated random walks and Lévy walks. It demonstrates that the two models are complementary rather than competing descriptions of movement pattern data and shows that correlated random walks are a part of the Lévy walk family. It follows from this that vast numbers of Lévy walkers could be hiding in plain sight.

Turchin^1^ argued that correlated random walks are the most influential and practicable approach to describing animal movement patterns. In these models, an individual's trajectory through space is typically regarded as being made up of a sequence of distinct, independent, randomly oriented ‘steps’. It has long been recognized that the transformation of an animal's continuous movement path into a broken line is necessarily arbitrary and that probability distributions of steps lengths and turning angles are model artifacts^1^. Nonetheless, for most researchers this shortcoming is not important on a practical level (smoothing out the simulation data will not really change anything) and that the ‘problem’ can be fixed using ‘continuous-time’ correlated random walk models in which velocities evolve as a Markovian process^2^,^3^,^4^. This position changed somewhat when it was realized that, as a result of auto-correlation of velocities, continuous-time correlated random walk models produce Lévy walk movement patterns^5^; an increasingly popular but controversial model of animal movement patterns^6^,^7^,^8^. Lévy walks comprise clusters of short step lengths with longer movements between them. This pattern is repeated across all scales with the resultant clusters creating fractal patterns that have no characteristic scale because the root-mean-square step-length is a divergent quantity. The distribution of step lengths has a power-law tail, *p_l_*(*l*) ~ *l*^−*μ*^ where 1 < *μ* < 3. Lévy walks are controversial, in part, because many early studies had wrongly ascribed Lévy walks to some species through the use of inappropriate statistical techniques^9^,^10^. More recently, however, studies have provided compelling evidence that many organisms (a diverse range of marine predators, honeybees, mussels, *Escherichia coli*, *T-cells*) display movement patterns that can be approximated by Lévy walks, with *μ* ≈ 2, and these have been attributed to the execution of an innate, advantageous searching strategy^11^,^12^,^13^,^14^,^15^,^16^,^17^,^18^. Continuous-time correlated random walks do, however, present different Lévy walks characteristics. First, because they are present only over timescales shorter than the velocity autocorrelation timescale. Over longer timescales when there is complete de-correlation of velocities, motion is normally diffusive. Consequently continuous-time correlated random walks are like *truncated* Lévy walks. Second, the Lévy (power-law) exponent, *μ*, is 4/3 and so different from the exponents found in many previous empirical studies which are typically around 2. Nonetheless, the theoretical ‘duality’ of continuous-time correlated random walks and Lévy walks with *μ* = 4/3 suggests that correlated random walks are also part of the Lévy family and that the binary arguments surrounding the ‘Levy flight foraging hypothesis' are misguided as organisms move in ways that are well approximated by various types of Lévy walks, correlated random walks being but one^5^. It is also significant because correlated random walk modellers and state-space modellers, who rely heavily on the correlated random walk framework, have not really embraced or understood the importance or usefulness of Lévy walks as models of animal movement patterns^6^,^7^. It remains to be seen, however, whether the ‘duality’ is realized in practice.

Here we demonstrate that the production of Lévy walk movement patterns by continuous-time correlated random walk models, i.e., by biologically realistic correlated random walk models, is not just a theoretical, counter-intuitive curiosity resulting from an artifact of correlated random walk modelling. High-resolution movement pattern data for *Tenebrio* *molitor* L. beetles are shown to be explained both by simple continuous-time correlated random models and by distributions of distances travelled between consecutive turns that have heavy power-law tails – the hallmark of Lévy walks.

We will show that the movement patterns of *Tenebrio* beetles are consistent with theoretical expectations for one of the simplest continuous-time correlated random walk models - the Langevin equation (an Ornstein-Uhlenbeck process for velocity), 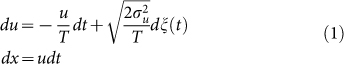
where *dξ*(*t*) is an incremental Wiener process with correlation property 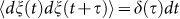. According to this model, velocities, *u*, are Gaussian distributed with mean zero and variance 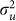 and are exponentially correlated on a timescale, *T*, i.e., 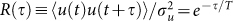 Furthermore, conditional velocity increments are Gaussian with mean and variance 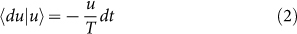


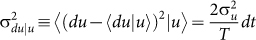
Mean-square displacements are described by 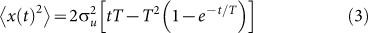
and are therefore ‘ballistic’ at short times (*t* < *T*) since 
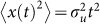
 and ‘diffusive’ at long-times because 
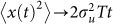
. The ballistic characteristic is typically associated with straight-line movements but can more generally be associated with Lévy walks^19^.

We will also show that the distances travelled by the *Tenebrio* beetles between successive changes in the direction of travel, hereafter referred to as ‘steps’, have distributions with *μ* = 4/3 power-scaling across a broad range of scales, as predicted theoretically^5^. This is indicative of Lévy walk movements with Lévy exponent *μ* = 4/3. We will thereby demonstrate that the duality of correlated-random-walks models and Lévy walks is realized in practice.

## Results

**Figure 1:**
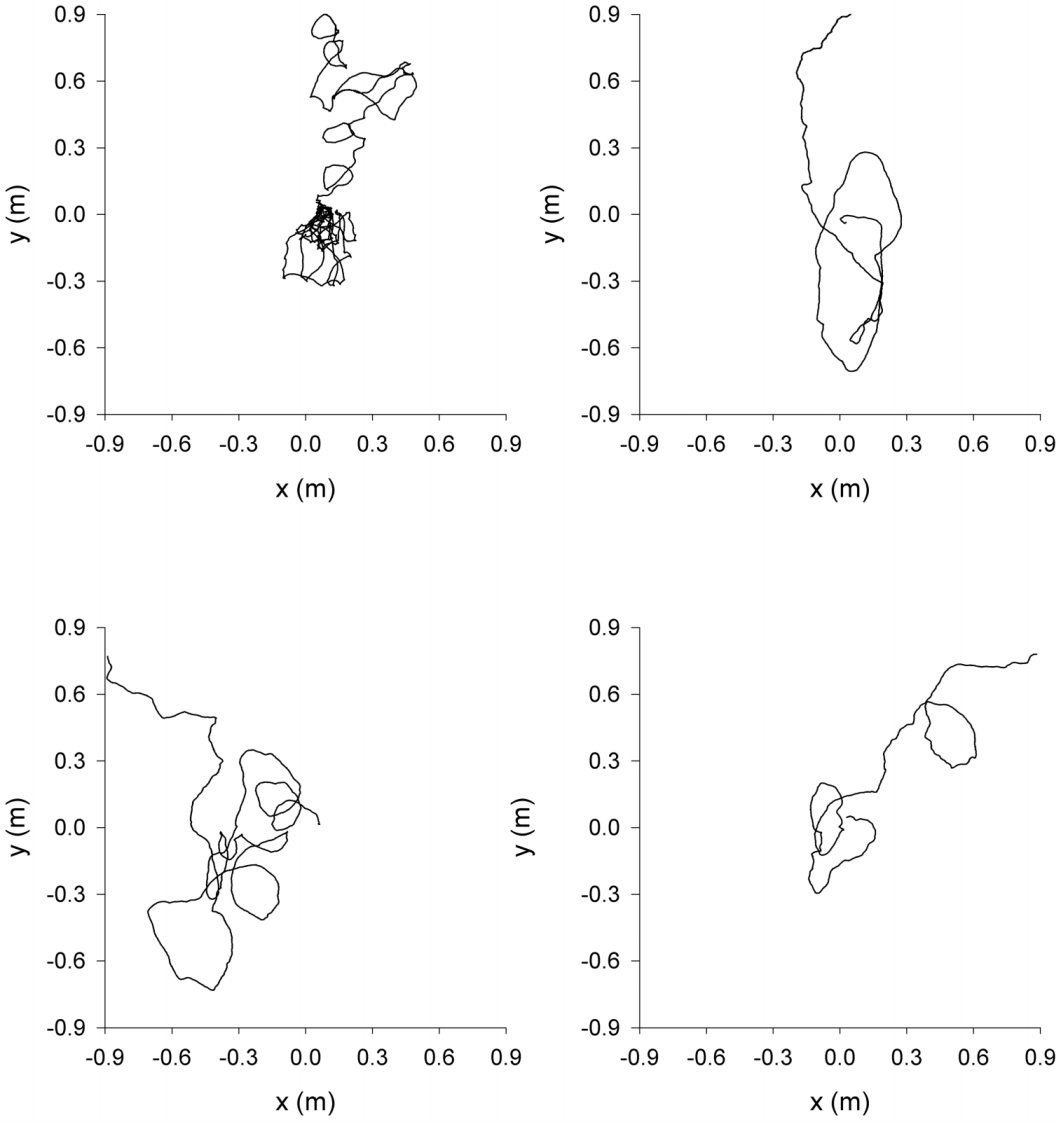
Four examples of non-orientated random movement patterns made by *Tenebrio* beetles within the experimental arena.

At the level of the individual *Tenebrio* beetle, different types of movement behavior were observed including near-straight-line ‘ballistic’ movements and looping, seemingly non-orientated, random movements (Fig. 1). Our attention was focused on the latter.

**Figure 2:**
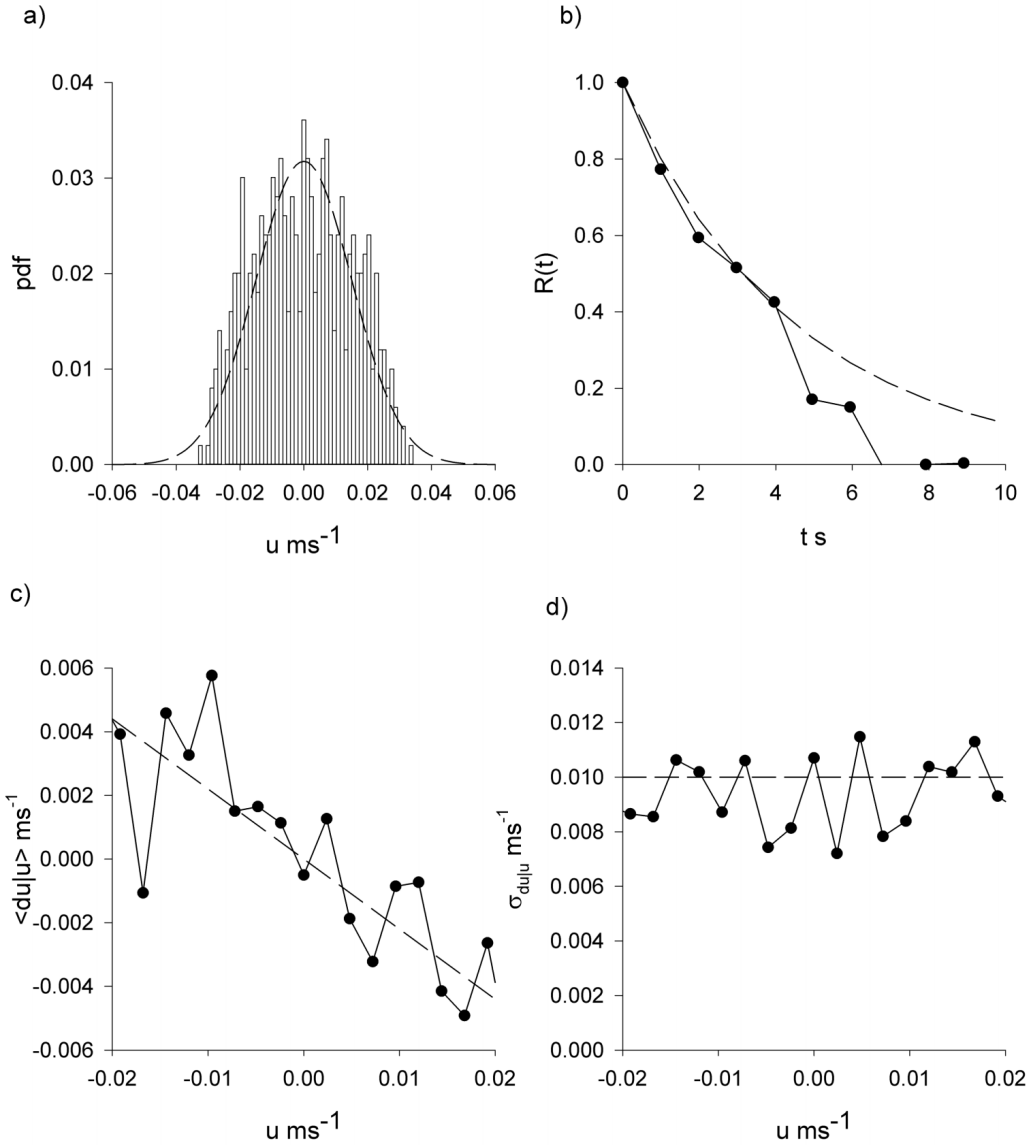
*Tenebrio* beetles have movement patterns that are well represented by the Langevin equation, Eq. (1). Analysis of the x-component of the first *Tenebrio* beetle track presented in Fig. 1. (a) Distribution of velocity (histogram). (b) The velocity autocorrelation function (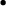). (c) The conditional mean velocity increments and (d) the associated conditional root-mean-square fluctuations (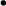). The connecting lines are added to guide the eye. Shown for comparison is a Gaussian with mean zero and equivalent variance, and other model expectations (dashed-lines).

**Figure 3:**
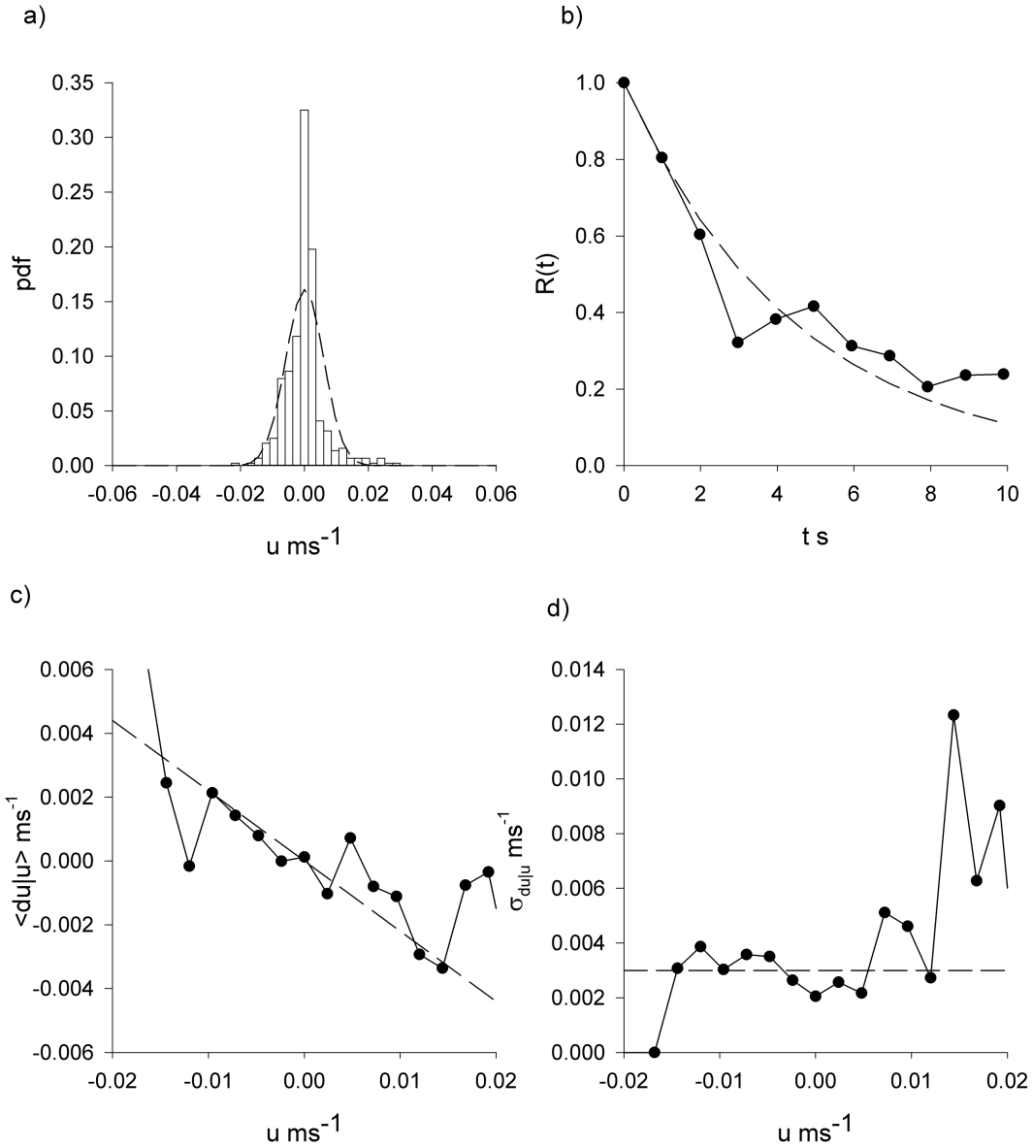
*Tenebrio* beetles have movement patterns that are well represented by the Langevin equation, Eq. (1). Analysis of the x-component of the second *Tenebrio* beetle track presented in Fig. 1. (a) Distribution of velocity (histogram). (b) The velocity autocorrelation function (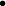). (c) The conditional mean velocity increments and (d) the associated conditional root-mean-square fluctuations (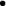). The connecting lines are added to guide the eye. Shown for comparison is a Gaussian with mean zero and equivalent variance, and other model expectations (dashed-lines).

**Figure 4:**
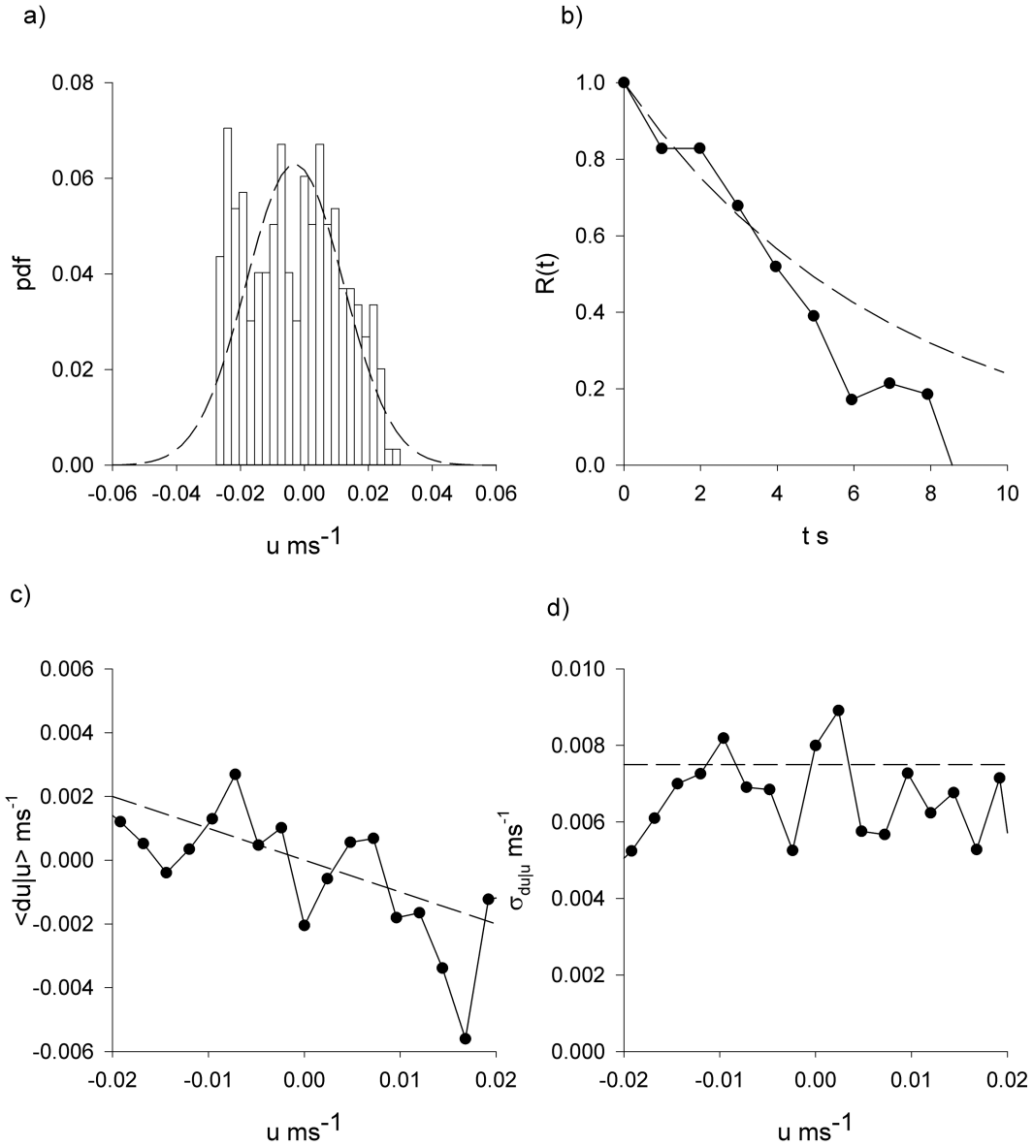
*Tenebrio* beetles have movement patterns that are well represented by the Langevin equation, Eq. (1). Analysis of the x-component of the third *Tenebrio* beetle track presented in Fig. 1. (a) Distribution of velocity (histogram). (b) The velocity autocorrelation function (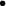). (c) The conditional mean velocity increments and (d) the associated conditional root-mean-square fluctuations (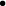). The connecting lines are added to guide the eye. Shown for comparison is a Gaussian with mean zero and equivalent variance, and other model expectations (dashed-lines).

**Figure 5:**
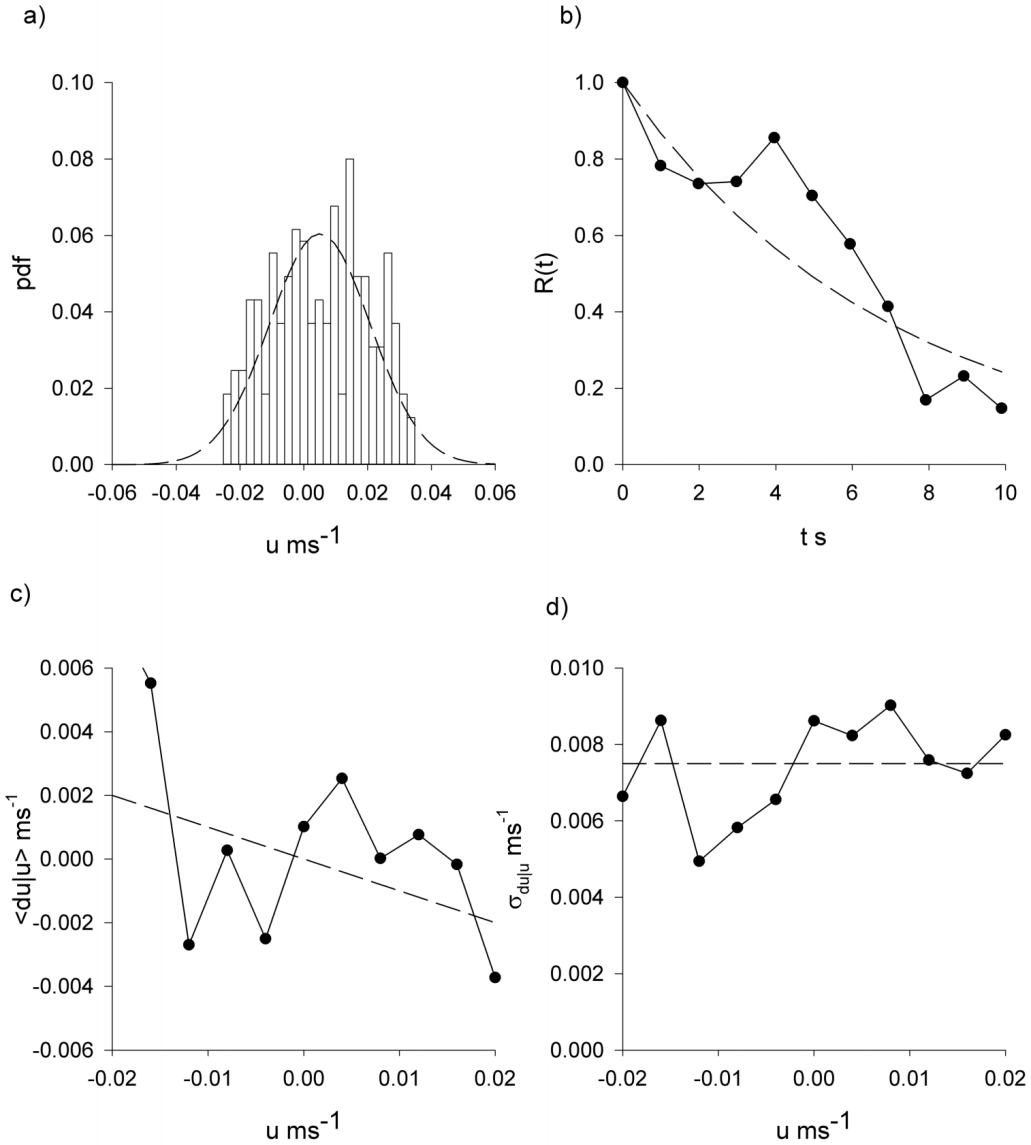
*Tenebrio* beetles have movement patterns that are well represented by the Langevin equation, Eq. (1). Analysis of the x-component of the fourth *Tenebrio* beetle track presented in Fig. 1. (a) Distribution of velocity (histogram). (b) The velocity autocorrelation function (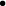). (c) The conditional mean velocity increments and (d) the associated conditional root-mean-square fluctuations (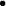). The connecting lines are added to guide the eye. Shown for comparison is a Gaussian with mean zero and equivalent variance, and other model expectations (dashed-lines).

**Figure 6:**
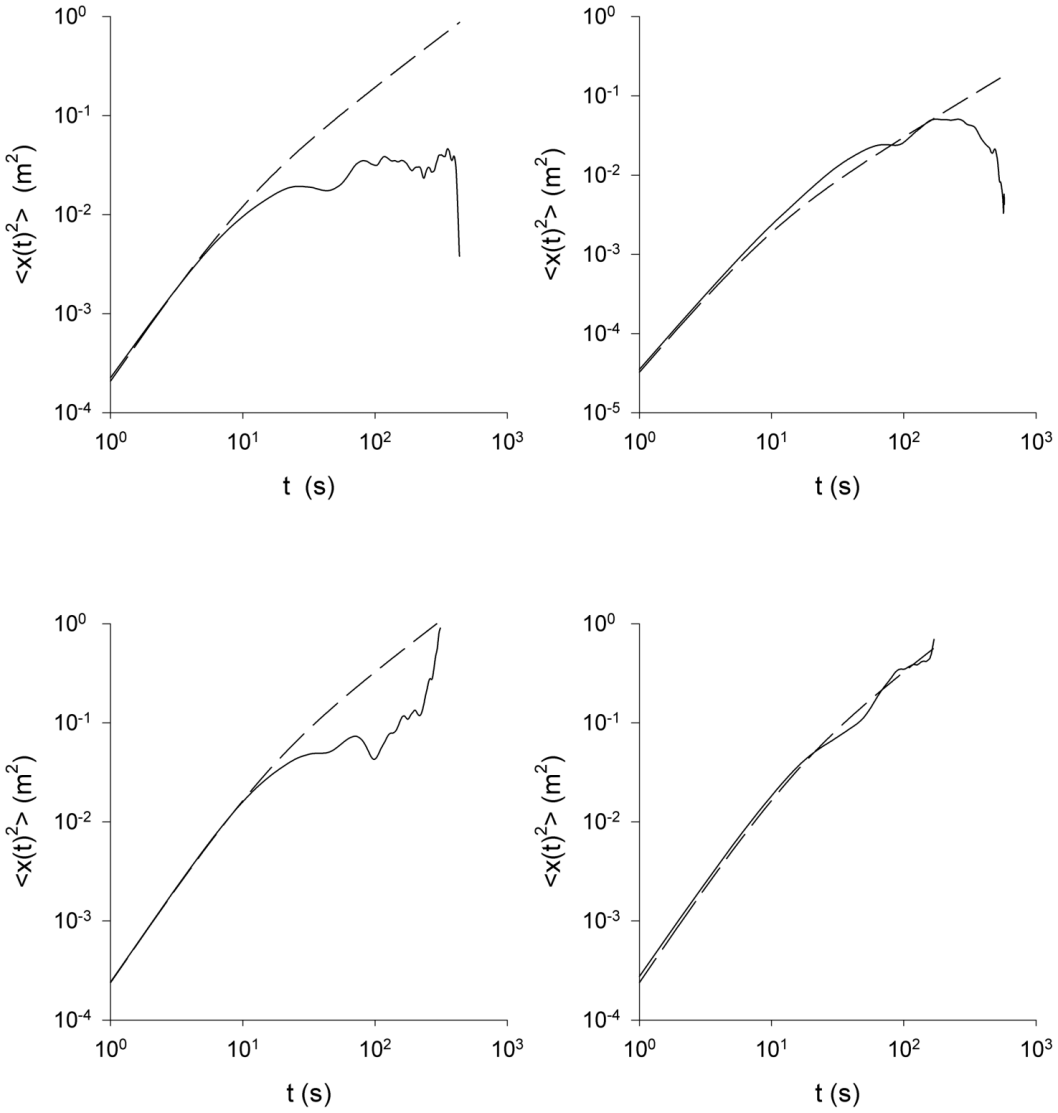
Integrated movements of the *Tenebrio* beetle are well represented by the Langevin equation, Eq. (1). Mean-squared displacements (x-components) for the four different *Tenebrio* beetle tracks presented in Fig. 1 (solid-lines). Shown for comparison are model expectations (dashed-lines). The velocity variances were calculated directly from the data, and the autocorrelation timescales were estimated from the velocity autocorrelation function.

In accordance with theoretical expectations, Eqn. 2, the *Tenebrio* beetle velocities are approximately Gaussian distributed and exponentially correlated, conditional mean velocity increments are proportional to velocity, and the associated conditional root-mean-square fluctuations appear to be independent of velocity (Figs. 2,3,4,5).The estimate for the correlation timescale, *T*, obtained from the velocity autocorrelation function (Figs. 2,3,4,5b), is consistent with the estimates obtained from the conditional mean velocity increment (Figs. 2,3,4,5c, Eqn. 2), and the associated conditional variance (Figs. 2,3,4,5d, Eqn. 2). Similarly, the velocity distribution and the conditional variances (Figs. 2,3,4,5d, Eqn. 2) provide consistent estimates for the velocity variance. The continuous-time correlated random walk model therefore provides an accurate, self-consistent description of the observations. Data are shown for one-component of the positions and velocities of four different beetles. Analogous results were obtained for the other direction of travel, and as a consequence the two-dimensional movement patterns can be accurately represented by two independent Langevin equations with individual-specific values of the model parameters, *σ_u_* and *T*. Mean-squared displacements made by the *Tenebrio* beetles are also consistent with the theoretical expectations, Eqn. 3, for the Langevin equation (Fig. 6).

**Figure 7:**
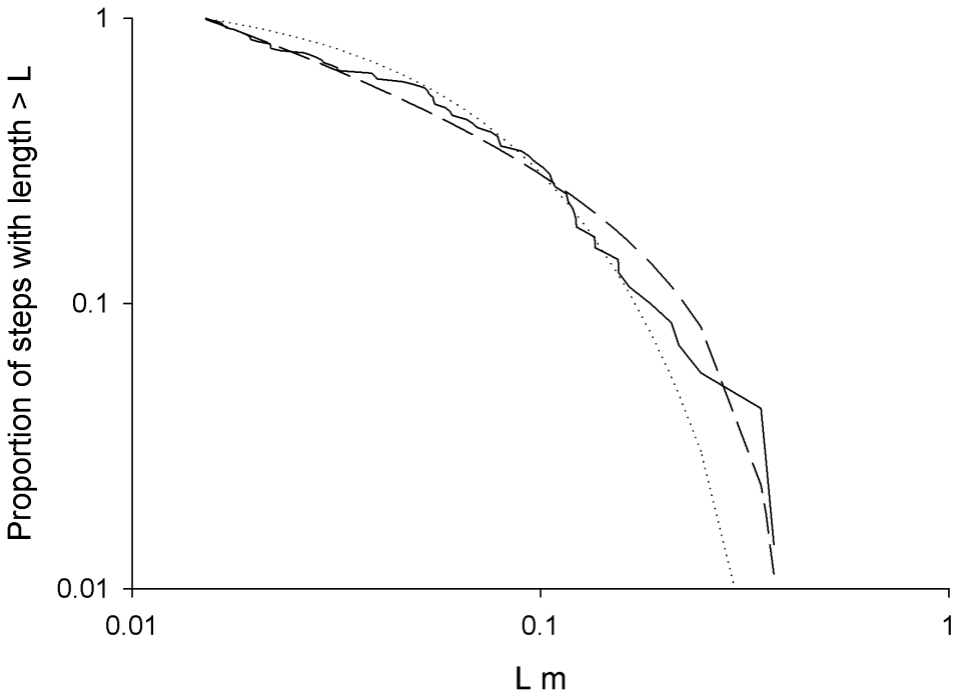
A demonstration that the key assertion that *Tenebrio* beetles have movement patterns that are well approximated as Lévy walks with *μ* = 4/3 is correct. Complement of the cumulative frequency distribution of the distances travelled by one *Tenebrio* beetle in the x-direction between consecutive turns (changes in the sign of the x-component of velocity) (solid-line), together with the best-fit power-law distribution (dashed-line) and best-fit exponential distribution (dotted-line). The best-fit distributions were obtained using maximum likelihood methods. The maximum likelihood estimate for the Lévy exponent is *μ* = 1.37, as expected.

**Figure 8:**
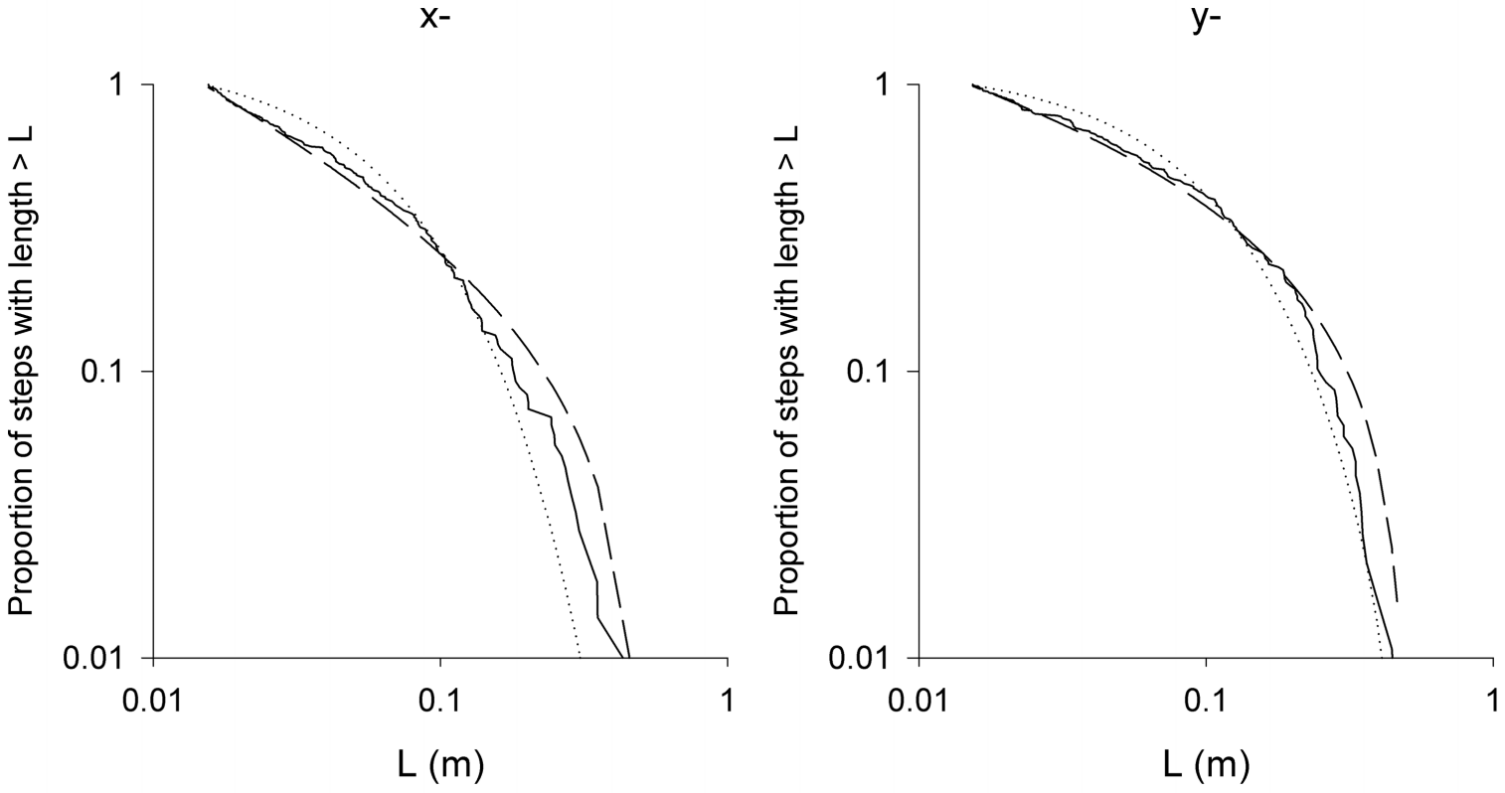
A further demonstration that *Tenebrio* beetle movement patterns are well approximated as Lévy walks with *μ* = 4/3. Complement of the cumulative frequency distribution of the distances travelled by *Tenebrio* beetles in the x- and y-directions between consecutive turns (changes in the sign of the x- and y-components of velocity) (solid-line), together with the best-fit power-law distribution (dashed-line) and best-fit exponential distribution (dotted-line). The best-fit distributions were obtained using maximum likelihood methods. Data has been pooled for 7 individuals. The maximum likelihood estimate for the Lévy exponent *μ* = 1.35, as expected.

The distances travelled in the x- direction between consecutive changes in the x- component of velocity show power-law scaling over an extended range of scales (Fig. 7). In accordance with theoretical expectations^5^, the maximum likelihood estimate for the Lévy exponent is *μ* = 1.37 and the Akaike weight for the power-law being the better model distribution is 0.96. Power-law scaling is evident for steps with length greater than the body-length, *1.5 cm*, of a Tenebrio beetle. Analogous results were found for the movements in the y- direction, and for other individuals (Fig. 8). This dual power-law scaling is indicative of the presence of 2-dimensional Lévy walk movement patterns^16^.

## Discussion

By dispensing with discretization, continuous-time correlated random walk models overcome inherent problems with discrete correlated random walk models in which movement patterns are represented by sequences of discrete steps whose statistical properties are model artifacts^1^. Nonetheless, randomness remains a ‘bug-bear’. “Of course, we do not know that animals truly move at random, like flipping coins to decide whether to turn right or left. Each individual could be a perfect automaton, rigidly reacting to environmental cues and its internal states in accordance with some set of behavioral rules. However, even if this were true, we might still choose to model behavior of such animals stochastically, because we would not have the perfect knowledge of all the deterministic rules driving these animals”^1^. Randomness would then be a modeling convention adopted because it is impractical, and not even helpful, to attempt to model individual movement deterministically. It can be effective because, in direct analogy with thermodynamic theory, even if animals do not move randomly, the collective behaviour of large numbers of such individuals may be indistinguishable (at the scale of the population) from what would result if they did^20^. This approach is aptly termed *behavioral minimalism*^21^. The production of Lévy walk movement patterns by continuous-time correlated random walk models would then be a model artifact akin to the distributions of step-lengths and turning-angles that characterise discrete correlated random walk models. Nonetheless, in animals with poorly developed sensory systems, like many insect herbivores, the impression of innate randomness is overwhelming, suggesting that for some of these organisms, Lévy walk movement patterns are an actuality. Here this was demonstrated to be the case for *Tenebrio* beetles.

The movement patterns of individual *Tenebrio* beetles in an unchanging and featureless arena, where external cues that can influence movement were limited, were found to be described by the Langevin equation; one of the simplest continuous-time correlated random walks. In accordance with a rigorous theoretical analysis^5^, *Tenebrio* beetle movement patterns were also found to have Lévy walk characteristics with Lévy exponent *μ* = 4/3. These Lévy walk characteristics extended over scales ranging from about the size of the beetles, 1.5 cm, to the maximum observed displacements from the release position, a distance of about 40 cm. These *μ* = 4/3 Lévy walk characteristics are a by-product of velocities being auto-correlated, as must be the case in actuality, and are not specific to the Langevin equation or to a requirement that velocities be Gaussian distributed. They may be without biological significance. Nonetheless, they suffice to show that correlated random walks are a part of the family of Lévy walks, that duality is observed in practice and that the utility of Lévy walks as models of animal movement pattern is not restricted to foraging, as is widely believed to be the case^22^. Their extent is determined by the autocorrelation timescale. We found, for example, that the direction of travel of *Pterostichus melanarius* (Illiger, 1798) and *Poecilus cupreus* Linnaeus, 1758 beetles tended to remain nearly constant in our experimental arena, so preventing the recording of any complex patterns of movements with multiple turns. This indicates that the autocorrelation timescales for these beetles was typically longer than the autocorrelation timescale for the *Tenebrio* beetles. Nonetheless, *Pterostichus* can perform Lévy walks in more complex but otherwise similar arenas^23^. Other animals can have very much longer autocorrelation timescales. Johnson et al.^24^, for example, reported that autocorrelation timescales of harbor seal (*Phoca vitulina*) and northern fur seal (*Callorhinus ursinus*) are several hours long. Lévy walk movement patterns on these scales should be evident in GPS tracking data, but have not been reported. Lévy walk movement patterns with *μ* = 1.25 (1.07, 1.43, 95%*CI*) and so consistent with model expectations have, however, been found in gray seals (*Halichoerus grypus*)^25^.

Nonetheless, the Lévy walk movements are not ubiquitous. Some *Tenebrio* beetles had circuitous movements akin to those made by some invertebrates, rodents, fish and even humans in the absence of external directional references^26^,^27^,^28^,^29^,^30^,^31^,^32^,^33^. In the *Tenebrio* beetles, the circuitous movements were found to be well represented by the Langevin equation if supplemented by an additional term, identified by Alt^3^,^4^, which induces a mean acceleration in a direction orthogonal to the direction of travel. This suggests that the circuitous movements seen in other taxa maybe also be captured by the modified Langevin equation and that a correlated-random-walk, Lévy walk duality should be evident in these taxa in the presence of detectable external directional references. It would be interesting to test for correlated-random-walk, Lévy walk duality more generally. We believe that this is likely to be found given the huge success that discrete correlated random walks have had as representations of non-orientated animal movement patterns. Continuous-time correlated random walks can fare no worse given that they are biologically more realistic. Indeed, a preliminary theoretical analysis shows that the duality of continuous-time correlated random walks and Lévy walks persists when there is switching between different modes, i.e., switching between different model parameter values, when the most probable speed is non-zero, and when movements are confined to a home range or subject to thigmotaxis. Strong evidence for Lévy walks may be in plain sight and could come from a re-analysis of correlated random walkers. Evidence may also be found in a re-analysis of collective movement patterns. The collective movements of fish schools, and so perhaps other animal groups, can, after all, be approximated by the Langevin equation^34^. These possibilities warrant further investigation.

## Methods

### Experimental cultures

The experimental *Tenebrio* were raised in stock cultures maintained at the University of Bourgogne and INRA Centre de Dijon. Cultures were fed *ad libitum* on a mixture of coarsely ground wheat flour and wheat husk, supplemented with sliced apple, and kept under ambient conditions of temperature (22 ± 1°C) and light for the duration of the experiment.

### Experimental design

The test surface consisted of clean, white cartridge paper (90 g m^−2^). Two 90 cm lengths were cut from a 90 cm wide roll and abutted on the floor of the laboratory to create an arena measuring 1.8 m × 1.8 m. A video camera (The Imaging Source DMK31AU03 USB2.0 camera with Computar T4Z2813CS-IR 2.8–12 mm F1.3 vari-focal lens) was mounted 3 m perpendicularly above the arena and the camera system adjusted so that the arena filled the camera frame. All natural sources of light were excluded for filming, which took place under red light and at ambient, room temperature (22 ± 1°C).

A single, replicate beetle was introduced to the center of the arena and left to acclimatize under a clear, plastic drinking cup for 3 minutes. Filming commenced once this cup was removed and stopped after 10 minutes or when the beetle touched a side of the arena and was therefore presumed to have left the experiment. 26 male and 29 female replicate beetles, selected at random, were used for the video experiments. The paper arena surface was changed between replicates.

The video was recorded to a Windows PC using IC Capture (v 3.0, The Imaging Source). We extracted the coordinates of beetle movement with time, from each replicate video, using the video-tracking system, Noldus EthoVision 7^35^,^36^.

### Data analysis

A first step in statistical tests for Lévy walks typically involves the discretisation of the movement pattern into a series of steps and turns. The identification of turning points, and therefore step-lengths, is, however, dependent on *ad-hoc* choices for the threshold turning angle. The problem can be avoided by projecting movement pattern data on to the x- and y-axes. This is because turning points in projected (one-dimensional) data can be identified unambiguously and occur only where there is a reversal in the direction travel (i.e., where the velocity changes sign) and because the projection preserves any Lévy walk characteristics^16^,^37^. This approach was used in an analysis of the vertical displacements of marine pelagic predators which provided strong support for Lévy walks^16^. We used this approach together with the Akaike information criterion^38^.The Akaike information criterion^38^ was used to test whether distributions of step-lengths in our empirical data are better represented as power-laws 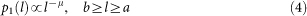
or as exponentials 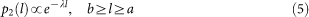
i.e., whether the movement patterns can be regarded as being an approximation to a Lévy walk or as an approximation to a Brownian walk. The Lévy exponent, *μ*, and the exponential decay rate, *λ*, were determined using log-maximum likelihood methods^39^. The start and end locations, *a* and *b*, of candidate power-law scaling behaviour was ascertained by visual inspection of the survival functions (the complement of the cumulative distribution functions). To construct the survival function, the simulation data for the step-lengths {*l_i_*} was first ranked from largest to smallest {*i* = 1…*n*}. The probability that a step-length is greater than or equal to *l_i_* (the survival function) was then estimated as *i*/*n*.The Akaike weights provide a measure of the relative fit of different models but do not necessarily imply that the favoured distribution is a good model for the data. Here absolute goodness-of-fits were gauged by visual inspection of the survival functions.
